# Systems Mapping for Hematopoietic Progenitor Cell Heterogeneity

**DOI:** 10.1371/journal.pone.0126937

**Published:** 2015-05-13

**Authors:** Linghua Zhou, Yong Shen, Libo Jiang, Danni Yin, Jingxin Guo, Hui Zheng, Hao Sun, Rongling Wu, Yunqian Guo

**Affiliations:** 1 Center for Computational Biology, Beijing Forestry University, Beijing, People’s Republic of China; 2 CAS Key Laboratory of Regenerative Biology, Guangdong Provincial Key Laboratory of Stem Cell and Regenerative Medicine, Guangzhou Institutes of Biomedicine and Health, Chinese Academy of Sciences, Guangzhou, Guangdong, People’s Republic of China; 3 Center for Statistical Genetics, Pennsylvania State University, Hershey, Pennsylvania, United States of America; The State University of New York at Stony Brook, UNITED STATES

## Abstract

Cells with the same genotype growing under the same conditions can show different phenotypes, which is known as “population heterogeneity”. The heterogeneity of hematopoietic progenitor cells has an effect on their differentiation potential and lineage choices. However, the genetic mechanisms governing population heterogeneity remain unclear. Here, we present a statistical model for mapping the quantitative trait locus (QTL) that affects hematopoietic cell heterogeneity. This strategy, termed systems mapping, integrates a system of differential equations into the framework for systems mapping, allowing hypotheses regarding the interplay between genetic actions and cell heterogeneity to be tested. A simulation approach based on cell heterogeneity dynamics has been designed to test the statistical properties of the model. This model not only considers the traditional QTLs, but also indicates the methylated QTLs that can illustrate non-genetic individual differences. It has significant implications for probing the molecular, genetic and epigenetic mechanisms of hematopoietic progenitor cell heterogeneity.

## Introduction

Cell fate decision is an important question during developmental processes, such as embryogenesis, neurogenesis, and hematopoiesis. During the hematopoiesis process, hematopoietic stem cells (HSCs) proliferate to self-renew or differentiate to progenitor cells, which generate mature blood cells. These progenitors, including common lymphoid progenitors (CLPs) and common myeloid progenitors (CMPs), can differentiate into more committed progenitors that give rise to blood cells. These progenitors can be used for bone marrow transplantation to treat diseases such as leukemia, sickle cell anemia, and thalassaemia [1–4].

Hematopoietic multipotential progenitors have two major differentiation choices: erythroid and myeloid lineages, which are regulated by the key transcription factors, Gata1 and PU.1. These two transcription factors positively regulate lineage-specific genes and repress each other [5]. In addition to transcriptional networks, genetic and epigenetic mechanisms are critical in determining cell fate.

Genetically identical hematopoietic progenitor cells growing under the same conditions can show differences in phenotypic characteristics, which is known as “population heterogeneity”, and has attracted interest for many years. However, it remains unclear whether this non-genetic characteristic affects cell fate determination. A previous report published in Nature by Chang et al. [6] showed that gene expression of noise controls the lineage choices of hematopoietic progenitor cells. However, the genetic mechanisms that control this process have not been explored in that paper. Cell fate conversions are dynamic with changes in chromatin structure regulated by DNA and histone modifications, including DNA methylation at symmetrical CG dinucleotides (CpG) and histone methylation and acetylation. Epigenetic regulation has been studied in hematopoietic lineage specification based on coordinated changes in gene expression, chromatin state, and DNA methylation [5,7,8].

Genetic mapping can provide a view of network and gene actions, as well as interactions with quantitative trait loci (QTLs), which can demonstrate the effects of genetic variation. Functional mapping developed by Wu et al. differs from the traditional mapping strategies, and is a very useful method to analyze dynamic data, as well as mapping QTLs related to development processes including cell apoptosis, cancer stem cell proliferation [9–12], et al. Clonal population heterogeneity, which is known as “non-genetic cell individuality”, cannot be analyzed using traditional QTLs. Several studies have examined the link between DNA methylation and gene expression, as well as mapping the methylated QTLs (meQTLs) to interpret the mechanisms underlying genetic variants [13,14]. meQTLs may increase our understanding of population heterogeneity and lineage choice problems, which cannot be demonstrated by alterations in the DNA sequences.

The aim of this article was to explore the genetic mechanisms regulating cell population heterogeneity and hematopoietic progenitor cell lineage choices. Besides the traditional QTL analysis, we mapped the effects of genetic variation on DNA methylation, focusing on mapping meQTLs that determine population heterogeneity and lineage choices.

## Methods

### Mathematical modeling of the evolution of two hematopoietic progenitor cell subpopulations

Hematopoietic progenitor cells show heterogeneity in one clonal population. The expression level of stem-cell-surface marker Sca-1 showed an approximately 1000-fold range within one newly derived clonal cell population based on flow cytometric analysis. Cells with the highest, middle and lowest ~15% Sca-1 expression level were isolated from one clonal population as separate subpopulations using fluorescence-activated cell sorting (FACS). Within hours, all three subpopulations showed narrow Sca-1 histograms; however, the three fractions regenerated Sca-1 histograms similar to that of the parental (unsorted) population after 21-day culture. A two-Gaussian model that best fitted the observed histogram evolution and restoration of the parental distribution was predominantly driven by state transitions between the subpopulations. Linear and nonlinear ordinary differential equations (ODEs) are used to describe the transition of the two subpopulations, respectively.

**Fig 1:**
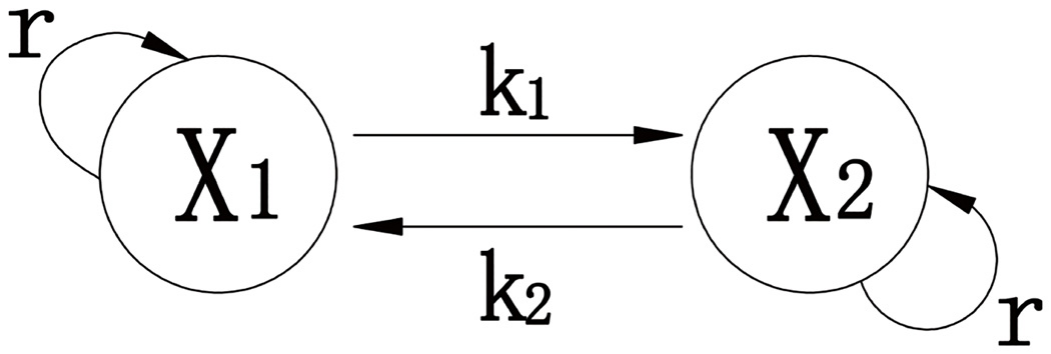
Linear model of two interacting and growing progenitor cell subpopulations.

Based on Fig 1, Chang et al. [6] proposed a linear model of equations for the size *x*_*i*_ of subpopulation *i*:

$$\begin{array}{l} \frac{dx_{1}}{dt}=rx_{1}-k_{1}x_{1}+k_{2}x_{2}\\ \frac{dx_{2}}{dt}=rx_{2}+k_{1}x_{1}-k_{2}x_{2} \end{array}$$ (1)

where *x*_*1*_ and *x*_*2*_ represent the sizes of subpopulations 1 and 2, *r* is the growth rate of both subpopulations, *k*_*1*_ is the transition rate from *x*_*1*_ to *x*_*2*_ and vice versa for *k*_*2*_.

**Fig 2:**
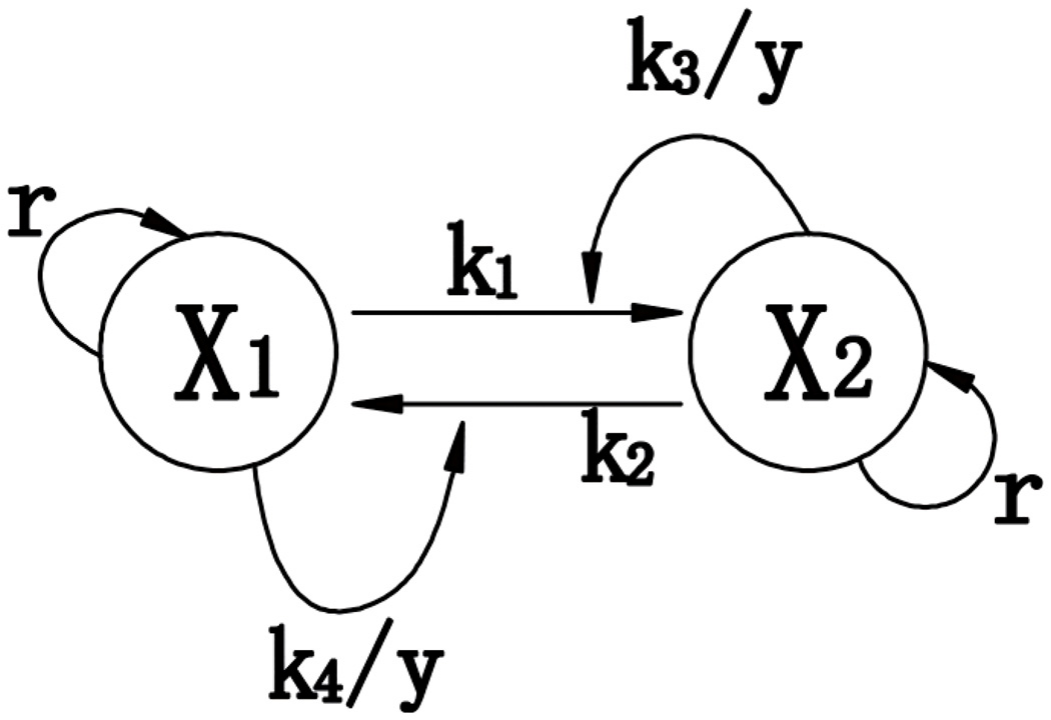
Nonlinear dynamic model of two interacting and growing progenitor cell subpopulations.

To increase our understanding of the asymptotic behavior and explain the sigmoidal increase of *x*_*1*_ and *x*_*2*_, a nonlinear dynamic model of two interacting and growing progenitor cell populations was proposed based on Fig 2. The ordinary differential equations governing the growth of the two subpopulations will then be:

$$\begin{array}{l} \frac{dx_{1}}{dt}=[rx_{1}-k_{1}x_{1}+k_{2}x_{2}]-k_{3}w_{2}x_{1}+k_{4}w_{1}x_{2}\\ \frac{dx_{2}}{dt}=[rx_{2}+k_{1}x_{1}-k_{2}x_{2}]+k_{3}w_{2}x_{1}-k_{4}w_{1}x_{2} \end{array}$$ (2)

where *x*_*1*_ and *x*_*2*_ represent the sizes of subpopulations 1 and 2, *r* is the growth rate of both subpopulations, *k*_*1*_ is the transition rate from *x*_*1*_ to *x*_*2*_ and vice versa for *k*_*2*_, and *k*_*3*_ and *k*_*4*_ are parameters determining the feedback rate.

### Statistical modeling of systems mapping

Systems mapping is a statistical model that views a complex phenotype as a dynamic system, dissects it into its underlying components, coordinates different components in terms of biological laws through mathematical equations, and maps specific genes that mediate each component and its connection with other components [15]. As a bottom—top model, a systems approach can identify specific QTLs that govern the developmental interactions between various components, giving rise to the function and behavior of the system. By estimating and testing mathematical parameters that specify the system, systems mapping allows us to predict the critical genetic mechanisms governing alterations in the physiological status of a phenotype.

### Sampling strategy

Suppose there is a natural population drawn at random. From a genetic perspective, we assume the original population follows Hardy-Weinberg equilibrium. The *n* sampled subjects are genotyped for a set of molecular markers, such as single nucleotide polymorphisms (SNPs). Assume that there exists a particular gene that controls the dynamics of cell interactions and growth. This gene has three alleles; namely, *Q*, *q*, and *q* ^*+*^, whose frequencies are *q*_*1*_, *q*_*2*,_ *and 1-q*_*1*_ *-q*_*2*_ in the population, and *q* ^*+*^ represents the methylated state of *q*. Let *μ*_*j*_ denote the genotypic value of size for *x*_*1*_ and *x*_*2*_ with *j* = 0 to 5 for *QQ*, *Qq*, *Qq* ^*+*^, *qq*, *qq* ^*+*^, and *q* ^*+*^ *q* ^*+*^, respectively.

The actual gene for cell dynamics, called the quantitative trait locus (QTL), cannot be observed directly, but it can be inferred from associated markers. Consider a SNP with alleles *M* vs. *m*, with respective frequencies *p*_*1*_ vs. 1—*p*_*1*_ in the population. The SNP and QTL form six haplotypes; namely, *MQ*, *Mq*, Mq^+^, *mQ*, *mq* and *mq* ^*+*^, with respective frequencies *P*_*MQ*_ *= P*_*M*_ *P*_*Q*_ *+ D*_*1*_,*P*_*Mq*_ *= P*_*M*_ *P*_*q*_+*D*_2,_ *$P_{Mq^{+}}=P_{M}P_{q}-D_{1}-D_{2}$*_,_ *P*_*mQ*_ *= P*_*m*_ *P*_*Q*_-*D*_1,_ *P*_*mq*_ *= P*_*m*_ *P*_*q*_-*D*_2,_ and $P_{mq^{+}}=P_{m}P_{q^{+}}+D_{1}+D_{2}$ in the population, where *D*_1_ and *D*_2_ are a linkage disequilibrium between the marker and QTL.

In the parental progeny of the population, the haplotypes unite randomly to generate 21 diplotypes and 18 genotypes, whose frequencies can be expressed in terms of haplotype frequencies. We express the genotype frequencies in matrix notation, with rows representing marker genotypes and columns representing QTL genotypes (Table 1). Thus, the conditional probabilities of QTL genotypes, conditional upon marker genotypes, can be derived according to Bayes’ theorem.

**Table 1:** Conditional probabilities of QTL genotypes given marker genotypes in a natural population.

	*QQ*	*Qq*	*Qq* ^*+*^	*qq*	*qq* ^*+*^	*q* ^*+*^ *q* ^*+*^
***MM***	*P*_*MQ*_^*2*^	*2P*_*MQ*_ *P*_*Mq*_	*2P*_*MQ*_ *P*_*Mq+*_	*P*_*Mq*_^*2*^	*2P*_*Mq*_ *P*_*Mq+*_	*P*_*Mq+*_^*2*^
***Mm***	*2P*_*MQ*_ *P*_*mQ*_	*2P*_*MQ*_ *P*_*mq*_ *+2P*_*Mq*_ *P*_*mQ*_	*2P*_*MQ*_ *P*_*mq+*_ *+2P*_*Mq*_ *+P*_*mQ*_	*2P*_*Mq*_ *P*_*mq*_	*2P*_*Mq*_ *P*_*mq+*_ *+2P*_*Mq+*_ *P*_*mQ*_	*2P*_*Mq+*_ *P*_*mq+*_
***mm***	*P*_*mQ*_^*2*^	*2P*_*mQ*_ *P*_*mq*_	*2P*_*mQ*_ *P*_*mq+*_	*P*_*mq*_^*2*^	*2P*_*mq*_ *P*_*mq+*_	*P*_*mq+*_^*2*^

#### Likelihood

Systems mapping embeds a system of differential equations into a genetic mapping setting constructed using a segregating population. Genetic mapping uses a mixture model-based likelihood to estimate genotype-specific parameters by assuming *j* QTL genotypes. For a progenitor cell population *i*, we obtain the size, *x*_*1*_ and *x*_*2*_, for the two following cell subpopulations at a finite set of times, *1*, …, *T*. A linear model for describing the phenotypic values of population *i* controlled by a putative gene is expressed as:

$$\begin{array}{l} {\text{x}}_{\text{1i}}(t)=\sum_{j=0}^{5}\xi_{i}\mu_{j}^{\left(x_{1}\right)}(t)+e_{i}^{\left(x_{1}\right)}\\ {\text{x}}_{\text{2i}}(t)=\sum_{j=0}^{5}\xi_{i}\mu_{j}^{\left(x_{2}\right)}(t)+e_{i}^{\left(x_{2}\right)} \end{array}$$ (3)

where *ξ*_*i*_ is an indicator variable defined as 1 if this population belongs to a specific genotype and 0 otherwise; time *t*, $\mu_{j}^{x_{1}}(t)$, and $\mu_{j}^{x_{2}}(t)$ are the genotypic values of type *k* for the cell size at time *t*, respectively; and $e_{i}^{x_{1}}$ and $e_{i}^{x_{2}}$ are the residual errors of subject *i* for cell *x*_*1*_ and *x*_*2*_ at time *t*, respectively.

For the mapping population of *n* members with marker information (*M*) and phenotypic data (*Y*_*i*_) for the two subpopulations, we formulate the likelihood as:

$$L(M,Y)=\prod_{i=1}^{n}\left[\sum_{j=0}^{5}\pi_{j|i}f_{j}(Y_{i};\Theta_{j},\Psi )\right]$$ (4)

whereY_*i*_ = (x_1*i*(1)_,…,x_1*i*(*T*),_x_2*i*(1)_,…,X_2*i*(*T*)_) represents phenotypic data for the subpopulation weights, *π*_*j|i*_ is the mixture proportion representing the conditional probability of QTL genotype *k* given the marker genotype of progenitor cell population *i*; and *f*_*j*_(*Y*_*i*_; Θ_*j*_, Ψ) is a multivariate normal distribution with an expected mean vector, $\mu_{j}=(\mu_{x_{1j(1)}};\ldots ;\mu_{x_{1j(T)}};\mu_{x_{2j(1)}};\ldots ;\mu_{x_{2j(T)}})$, for population *i* that belongs to QTL genotype *j*, and covariance matrix

$$\Sigma =\left(\begin{array}{cc} \Sigma_{1} & \Sigma_{1\times 2}\\ \Sigma_{2\times 1} & \Sigma_{2} \end{array}\right)$$ (5)

with Σ_1_and Σ_2_ being the time-dependent covariance matrix for *x*_*1*_ and *x*_*2*_, respectively, and Σ_1x2_ = Σ_2×1_^T^ being the time-dependent covariance matrix between these two variables.

#### Modeling mean vectors

The biological merit of systems mapping is to model genotype-specific differences in the time-dependent mean vector using a biologically meaningful mathematical equation [9]. When modeling the dynamic behavior of progenitor cell transition, Chang et al. [6] developed a set of ordinary differential equations (ODEs) as shown in (1) and (2). The overall behavior of progenitor cell heterogeneity can be described and quantified by ODE solution's parameters (*r*, *k*_*1*_, *k*_*2*_) for the linear model and parameters (*r*, *k*_*1*_, *k*_*2*_, *k*_*3*_, *k*_*4*_) for the nonlinear model. Differences in any one or more of these parameters will influence cell transition dynamics.

For a particular QTL genotype *j*, the dynamic behavior of ODE can be specified by a set of parameters Θ_*uj*_ = (*r*_*j*_,*k*_*1j*_,*k*_*2j*_) (for the linear differential equation) or Θ_*uj*_ = (*r*_*j*_,*k*_*1j*_,*k*_*2j*,_ *K*_*3j*,_ *k*_*4j*_) (for nonlinear differential equations). In other words, by changing any one or more parameters in Θ_*uj*_, the trajectory of cell transition dynamics may change. Thus, by incorporating ODE into functional mapping, a statistical framework originally derived to estimate QTL genotype-specific ODE parameters, Θ_*uj*_, can be used to assess the genetic effects of QTLs on progenitor cell transition by comparing the genotypic differences of the parameters.

#### Modeling covariance structure

The statistical power of systems mapping is partly the result of structured modeling of the covariance matrix. There are many approaches available to model the covariance structure, including autoregressive [9], antedependent [16], autoregressive moving average [17], nonparametric [18], and semiparametric [19]. These approaches have their own advantages and disadvantages in terms of efficiency, flexibility, and parsimony. Of these approaches, the antedependent model shows a good balance between these properties, and Zhao et al. [20] extended it to model the covariance structure of multiple variables. In this article, we use a bivariate antedependence approach for modeling the structure of the covariance matrix (5).

The residual errors of the linear model (3) at time *t* depend on the previous residuals with the degree of dependence decaying with time lag. If the current residuals only depend on their first preceding ones, the model is called a first-order antedependence model [SAD(1)]. The SAD(1) model specifies time-dependent residual errors for population *i* as:

$$\begin{array}{l} e_{i}^{\left(1\right)}(t)=\varphi_{1}e_{i}^{\left(1\right)}(t-1)+\phi_{1}e_{i}^{\left(1\right)}(t-1)+\varepsilon_{i}^{\left(1\right)}(t)\\ e_{i}^{\left(2\right)}(t)=\varphi_{2}e_{i}^{\left(2\right)}(t-1)+\phi_{2}e_{i}^{\left(2\right)}(t-1)+\varepsilon_{i}^{\left(2\right)}(t) \end{array}$$ (6)

where *ϕ*_1_ (or *φ*_2_) and *φ*_*1*_ (or *ϕ*_2_) are the unrestricted antedependence parameters induced by variable 1 (or 2) itself and by the other variable 2 (or 1), respectively; $\varepsilon_{i}^{1}(t)$ and $\varepsilon_{i}^{2}(t)$ are the ‘‘innovation” errors assumed to be bivariate-normally distributed with mean zero and variance matrix:

$$\Sigma_{\varepsilon }(t)=\left(\begin{array}{cc} \upsilon_{1}^{2}(t) & \upsilon_{1}(t)\upsilon_{2}(t)\rho (t)\\ \upsilon_{1}(t)\upsilon_{2}(t)\rho (t) & \upsilon_{2}^{2}(t) \end{array}\right)$$ (7)

For simplicity, we assume that Σ_*ε*_(*t*) is time-independent; i.e., *ν*_1_, *ν*_2_, and *ρ* are not dependent on time. Based on the above assumptions, Σ_*ε*_ becomes a block matrix with the diagonal repeating the following unit *T* times,

$$\Sigma_{\varepsilon }=\left(\begin{array}{cc} \upsilon_{1}^{2} & \upsilon_{1}\upsilon_{2}\rho \\ \upsilon_{1}\upsilon_{2}\rho & \upsilon_{2}^{2} \end{array}\right)$$ (8)

All parameters modeling the covariance matrix Σ_ε_ are arrayed in vector Ψ_*e*_ = (*ϕ*_1_,*φ*_2_,*φ*_*1*_,*ϕ*_2_,*υ*_1_,*υ*_2_,*ρ*). With model (6), closed forms for the determinant and inverse of the structured matrix, which enhances computing efficiency, were derived [20].

#### Computational algorithms

Three algorithms were integrated to estimate marker-QTL haplotype frequencies using the EM algorithm; the genotype-specific ODE parameters for progenitor cell transition dynamics by the fourth-order Runge-Kutta algorithm [21,22] and the SAD (1) parameters for the covariance structure using simplex algorithm. We derived a closed-form solution for the EM algorithm to estimate haplotype frequencies. In the E step, we calculated the posterior probability with which population *i* carries QTL mating type genotype *j*_*m*_ using:

$$\Pi_{j|i}=\frac{\pi_{j|i}f_{j}(Y_{i})}{\sum_{j=0}^{5}\pi_{j|i}f_{j}(Y_{i})}$$ (9)

In the M step, the calculated posterior probability was used to estimate marker-QTL haplotype frequencies by:

$$\begin{array}{l} {\overset{\wedge }{P}}_{MQ}=\frac{1}{2n}(2\Pi_{5|2i}+\Pi_{4|2i}+\Pi_{3|2i}+\Pi_{5|1i}+\theta_{1}\Pi_{4|1i}+\theta_{2}\Pi_{3|1i})\\ {\overset{\wedge }{P}}_{Mq}=\frac{1}{2n}(2\Pi_{2|2i}+\Pi_{4|2i}+\Pi_{1|2i}+\Pi_{2|1i}+(1-\theta_{1})\Pi_{4|1i}+\theta_{3}\Pi_{1|1i})\\ {\overset{\wedge }{P}}_{Mq^{+}}=\frac{1}{2n}(2\Pi_{0|2i}+\Pi_{3|2i}+\Pi_{1|2i}+\Pi_{0|1i}+(1-\theta_{2})\Pi_{3|1i}+(1-\theta_{3})\Pi_{1|1i})\\ {\overset{\wedge }{P}}_{mQ}=\frac{1}{2n}(2\Pi_{5|0i}+\Pi_{5|1i}+\Pi_{4|0i}+\Pi_{3|0i}+(1-\theta_{1})\Pi_{4|1i}+(1-\theta_{2})\Pi_{3|1i})\\ {\overset{\wedge }{P}}_{mq}=\frac{1}{2n}(2\Pi_{2|0i}+\Pi_{2|1i}+\Pi_{4|0i}+\Pi_{1|0i}+\theta_{1}\Pi_{4|1i}+(1-\theta_{3})\Pi_{1|1i})\\ {\overset{\wedge }{P}}_{mq^{+}}=\frac{1}{2n}(2\Pi_{0|0i}+\Pi_{0|1i}+\Pi_{3|0i}+\Pi_{1|0i}+\theta_{2}\Pi_{3|1i}+\theta_{3}\Pi_{1|1i}) \end{array}$$

$$\begin{array}{l} \theta_{1}=P_{MQ}P_{mq}/(P_{MQ}P_{mq}+P_{Mq}P_{mQ})\\ \theta_{2}=P_{MQ}P_{mq^{+}}/(P_{MQ}P_{mq^{+}}+P_{Mq^{+}}P_{mQ})\\ \theta_{3}=P_{Mq}P_{mq^{+}}/(P_{Mq}P_{mq^{+}}+P_{Mq^{+}}P_{mq}) \end{array}$$ (10)

In each iteration of the EM step, we estimated ODE parameters, $\Theta_{u_{j}}=(r_{j},k_{1j},k_{2j})$(for the linear differential equation) or $\Theta_{u_{j}}=(r_{j},k_{1j},k_{2j},k_{3j},k_{4j})$ (for nonlinear differential equations) using the Runge-Kutta algorithm, and covariance-structuring parameters, and Ψ_*e*_ = (*ϕ*_1_,*φ*_2_,*φ*_*1*_,*ϕ*_2_,*υ*_1_,*υ*_2_,*ρ*), using the simplex algorithm. The Runge—Kutta and simplex algorithms are integrated within the EM framework to generate each iterative procedure, which is repeated until the parameters converge to stable values.

### Hypothesis tests

Whether there are specific QTLs for progenitor cell transition dynamics can be tested by formulating the following hypotheses:

$${\text{H}}_{0}:\;\Psi_{\mu_{jm}}\equiv \Psi_{\mu }\;j=\;5,4,3,2,\;1,\;0;\;m=\;2,\;1,\;0$$ (11)

$${\text{H}}_{1}:\text{ At least one of the equalities above does not hold,}$$

where the H_0_ corresponds to the reduced model, in which only one single curve of transition dynamics exists; and H_1_ corresponds to the full model, in which there exists six such different curves to fit the data. We calculate this hypothesis to reduce the full model using the log-likelihood ratio (LR). The critical threshold is determined empirically from permutation tests [23].

Two variables, *x*_*1*_ and *x*_*2*_, may or may not be controlled by the same gene. The pleiotropic control of the gene over these two variables can be tested by formulating the null hypotheses: H_0_:r_*jm*_ ≡ r and k_*ijm*_ ≡ *k*_i_ j = 5,4,3,2, 1, 0; m = 2, 1, 0; i = 1,2 for the linear model and 1,2,3,4 for the nonlinear model

$${\text{H}}_{1}:\text{ At least one of the equalities above does not hold}$$ (12)

If the null hypothesis is rejected, this indicates that the gene detected exerts a pleiotropic effect on progenitor cell heterogeneity.

## Results

We performed simulation experiments to examine the statistical properties of the model built for genetic mapping of the two subpopulations of hematopoietic progenitor cell transitions. Fu et al. provided much more detailed information regarding the test and validation of systems mapping [24]. The transition process was described by linear and nonlinear ODEs, and the simulation assumed different heritabilities (0.05, 0.1 and 0.2) under different human population sizes (200 and 400) at Hardy-Weinberg equilibrium, considering a SNP marker that is associated with a putative QTL(with six genotypes *QQ*, *Qq*, *Qq* ^*+*^, *qq*, *qq* ^*+*^, and *q* ^*+*^ *q* ^*+*^) through linkage disequilibrium (LD). The transition parameters $\Theta_{u_{j}}$ = (*r*_*j*_, *k*_*1j*_, *k*_*2j*_) and $\Theta_{u_{j}}$ = (*r*_*j*_, *k*_*1j*_, *k*_*2j*_, *k*_*3j*_, *k*_*4j*_) for the six genotypes were determined in the ranges of empirical estimates of these parameters. Note that for computational simplicity, *r*, *k*_*1*_, *k*_*2*_, *k*_*3*_ and *k*_*4*_ are provided as shown in Tables 2, 3, 4 and 5. Based on allele frequencies of the marker, QTL, and their LD, joint marker and phenotypic data were simulated. The genetic parameters (*P*, *Q*_*1*_, *Q*_*2*_, *D*_*1*_, and *D*_*2*_) of the QTL can be estimated with high precision using the EM algorithm. The genotype-specific mean vectors were modeled based on ODE's solution (3) where the covariance matrix was structured by the first-order antedependence model [SAD(1)] with correlation and variance parameters, Ψ_*e*_ = (*ϕ*_1_,*φ*_2_,*φ*_*1*_,*ϕ*_2_,*υ*_1_,*υ*_2_,*ρ*), for *w*_*1*_ and *w*_*2*_.

**Table 2:** MLEs of linear ODE parameters that define the dynamics of cell transition for six different QTL genotypes and the association between the marker and QTL in a natural population of 200 based on different assumptions of heritability of the simulated QTL.

Cell Transition Parameters																							
		QQ					Qq					Qq+			qq			qq+			q+q+		
		Given			MLE(Std.)		Given		MLE(Std.)			Given	MLE(Std.)		Given	MLE(Std.)		Given		MLE(Std.)	Given		MLE(Std.)
Heritability of the simulated QTL is *H* ^*2*^ *=* 0.05																							
*r*			0.12			0.1184(0.0053)	0.15		0.1502(0.0023)	0.24			0.2396(0.0027)		0.2	0.1993(0.0019)		0.27		0.2707(0.0020)	0.3		0.3045(0.0163)
*k*_*1*_			0.003			0.0045(0.0011)	0.027		0.0307(0.0017)	0.05			0.0637(0.0093)		0.015	0.0167(0.0013)		0.018		0.0197(0.0016)	0.011		0.0133(0.0027)
*k*_*2*_			0.006			0.0075(0.0028)	0.0011		0.0014(0.0012)	0.0007			0.0009(0.0002)		0.0009	0.0010(0.0002)		0.007		0.0072(0.0018)	0.003		0.0035(0.0011)
Heritability of the simulated QTL is *H* ^*2*^ *=* 0.1																							
*r*			0.12			0.1165(0.0039)	0.15		0.1504(0.0016)	0.24			0.2396(0.0024)		0.2	0.1995(0.0017)		0.27		0.2706(0.0014)	0.3		0.3042(0.0129)
*k*_*1*_			0.003			0.0046(0.0010)	0.027		0.0307(0.0016)	0.05			0.0607(0.0087)		0.015	0.0164(0.0001)		0.018		0.0194(0.0011)	0.011		0.0127(0.0030)
*k*_*2*_			0.006			0.0075(0.0020)	0.0011		0.0014(0.0008)	0.0007			0.0009(0.0002)		0.0009	0.0010(0.0002)		0.007		0.0072(0.0015)	0.003		0.0035(0.0009)
Heritability of the simulated QTL is *H* ^*2*^ *=* 0.2																							
*r*			0.12			0.1175(0.0031)	0.15		0.1504(0.0015)	0.24			0.2397(0.0023)		0.2	0.1996(0.0011)		0.27		0.2703(0.0012)	0.3		0.3018(0.0070)
*k*_*1*_			0.003			0.0044(0.0010)	0.027		0.0299(0.0016)	0.05			0.0572(0.0074)		0.015	0.0162(0.0008)		0.018		0.0194(0.0009)	0.011		0.013(0.0019)
*k*_*2*_			0.006			0.0073(0.0019)	0.0011		0.0014(0.0007)	0.0007			0.0008(0.0002)		0.0009	0.0010(0.0001)		0.007		0.0071(0.0013)	0.003		0.0033(0.0009)
**Genetic Parameters**																							
	*H* ^*2*^ *=* 0.05							*H* ^*2*^ *=* 0.1						*H* ^*2*^ *=* 0.2									
	Given			MLE(Std.)				Given			MLE(Std.)			Given			MLE(Std.)						
*P*	0.6			0.6062(0.0216)				0.6			0.5962(0.0295)			0.6			0.5976(0.0243)						
*Q*_*1*_	0.4			0.3972(0.0211)				0.4			0.4026(0.0218)			0.4			0.4008(0.0251)						
*Q*_*2*_	0.5			0.5036(0.0223)				0.5			0.4963(0.0231)			0.5			0.4973(0.0272)						
*D*_*1*_	0.01			0.0152(0.0191)				0.01			0.0104(0.0177)			0.01			0.0114(0.0182)						
*D*_*2*_	0.02			0.0146(0.0213)				0.02			0.0172(0.0175)			0.02			0.0213(0.0203)						
**Matrix Structuring Parameters**																							
	*H* ^*2*^ *=* 0.05							*H* ^*2*^ *=* 0.1						*H* ^*2*^ *=* 0.2									
	Given			MLE(Std.)				Given			MLE(Std.)			Given			MLE(Std.)						
*ϕ*_*1*_	0.76			0.7644(0.0387)				0.7			0.7204(0.0424)			0.6			0.6004(0.0362)						
*ϕ*_*2*_	0.62			0.6238(0.0321)				0.564			0.5568(0.0392)			0.47			0.4694(0.0362)						
*φ*_*1*_	1.22			1.2194(0.0351)				1.24			1.2412(0.0288)			1.25			1.2569(0.0329)						
*φ*_*2*_	1.23			1.2279(0.0249)				1.21			1.2134(0.0298)			1.2			1.1992(0.0245)						
*ν*_*1*_	0.01			0.0112(0.0031)				0.01			0.0111(0.0018)			0.01			0.0108(0.0017)						
*ν*_*2*_	0.01			0.0106(0.0052)				0.01			0.0101(0.0027)			0.01			0.0103(0.0024)						
*ρ*	0.4824			0.4849(0.0681)				0.4824			0.4809(0.0642)			0.4824			0.4891(0.4525)						

Std. represents the standard deviation of the estimates obtained from 100 simulation replicates.

**Table 3:** MLEs of linear ODE parameters that define the dynamics of cell transition for six different QTL genotypes and the association between the marker and QTL in a natural population of 400 based on different assumptions of heritability of the simulated QTL.

Cell Transition Parameters																				
		QQ			Qq				Qq+			qq			qq+			q+q+		
		Given		MLE(Std.)	Given		MLE(Std.)		Given	MLE(Std.)		Given		MLE(Std.)	Given		MLE(Std.)	Given		MLE(Std.)
Heritability of the simulated QTL is *H* ^*2*^ *=* 0.05																				
*r*		0.12		0.1197(0.0054)	0.15		0.1493(0.002)		0.24	0.2401(0.0032)		0.2		0.1993(0.0021)	0.27		0.2713(0.0019)	0.3		0.3024(0.0049)
*k*_*1*_		0.003		0.0047(0.0010)	0.027		0.0308(0.0013)		0.05	0.065(0.0101)		0.015		0.0163(0.0012)	0.018		0.0197(0.0013)	0.011		0.0135(0.0019)
*k*_*2*_		0.006		0.0087(0.0019)	0.0011		0.0014(0.0002)		0.0007	0.0010(0.0002)		0.0009		0.0010(0.0002)	0.007		0.0073(0.0017)	0.003		0.0035(0.0010)
Heritability of the simulated QTL is *H* ^*2*^ *=* 0.1																				
*r*		0.12		0.1165(0.0042)	0.15		0.1507(0.0016)		0.24	0.2399(0.0027)		0.2		0.1994(0.0013)	0.27		0.2708(0.0012)	0.3		0.3016(0.0060)
*k*_*1*_		0.003		0.0047(0.0010)	0.027		0.0308(0.0014)		0.05	0.063(0.0067)		0.015		0.0166(0.0009)	0.018		0.0196(0.0011)	0.011		0.013(0.002)
*k*_*2*_		0.006		0.0076(0.00018)	0.0011		0.0015(0.0002)		0.0007	0.0009(0.0002)		0.0009		0.0010(0.0002)	0.007		0.0072(0.0013)	0.003		0.0035(0.0009)
Heritability of the simulated QTL is *H* ^*2*^ *=* 0.2																				
*r*		0.12		0.1167(0.0027)	0.15		0.1504(0.0013)		0.24	0.2397(0.0022)		0.2		0.1995(0.0011)	0.27		0.2707(0.0011)	0.3		0.3013(0.0036)
*k*_*1*_		0.003		0.0046(0.0008)	0.027		0.0305(0.0013)		0.05	0.060(0.0062)		0.015		0.0165(0.0007)	0.018		0.0194(0.0008)	0.011		0.0135(0.0017)
*k*_*2*_		0.006		0.0069(0.0016)	0.0011		0.0015(0.0001)		0.0007	0.0009(0.0001)		0.0009		0.0010(0.0001)	0.007		0.0073(0.0009)	0.003		0.0035(0.0008)
**Genetic Parameters**																				
	*H* ^*2*^ *=* 0.05					*H* ^*2*^ *=* 0.1					*H* ^*2*^ *=* 0.2									
	Given		MLE(Std.)			Given		MLE(Std.)			Given		MLE(Std.)							
*P*	0.6		0.6000(0.0172)			0.6		0.6006(0.0196)			0.6		0.5992(0.0134)							
*Q*_*1*_	0.4		0.3953(0.021)			0.4		0.3979(0.0171)			0.4		0.402(0.0173)							
*Q*_*2*_	0.5		0.5048(0.0192)			0.5		0.503(0.0169)			0.5		0.5011(0.0147)							
*D*_*1*_	0.01		0.0111(0.0097)			0.01		0.0111(0.0122)			0.01		0.0109(0.0109)							
*D*_*2*_	0.02		0.0198(0.0103)			0.02		0.0171(0.0117)			0.02		0.0188(0.0112)							
**Matrix Structuring Parameters**																				
	*H* ^*2*^ *=* 0.05					*H* ^*2*^ *=* 0.1					*H* ^*2*^ *=* 0.2									
	Given		MLE(Std.)			Given		MLE(Std.)			Given		MLE(Std.)							
*ϕ*_*1*_	0.76		0.7566(0.0513)			0.7		0.6947(0.0478)			0.6		0.5853(0.0418)							
*ϕ*_*2*_	0.62		0.6102(0.0501)			0.564		0.5375(0.0386)			0.47		0.4532(0.0341)							
*φ*_*1*_	1.22		1.2338(0.0371)			1.24		1.2561(0.0336)			1.25		1.2605(0.0293)							
*φ*_*2*_	1.23		1.2396(0.0310)			1.21		1.2385(0.0356)			1.2		1.2216(0.0337)							
*ν*_*1*_	0.01		0.0120(0.002)			0.01		0.0111(0.0017)			0.01		0.0105(0.0013)							
*ν*_*2*_	0.01		0.0114(0.0043)			0.01		0.0103(0.0014)			0.01		0.0101(0.0011)							
*ρ*	0.4824		0.4136(0.0477)			0.4824		0.4304(0.0393)			0.4824		0.4503(0.0377)							

Std. represents the standard deviation of the estimates obtained from 100 simulation replicates.

**Table 4:** MLEs of nonlinear ODE parameters that define the dynamics of cell transition for six different QTL genotypes and the association between the marker and QTL in a natural population of 200 based on different assumptions of heritability of the simulated QTL.

Cell Transition Parameters																				
	QQ				Qq			Qq+			qq				qq+			q+q+		
	Given		MLE(Std.)		Given	MLE(Std.)		Given		MLE(Std.)	Given		MLE(Std.)		Given		MLE(Std.)	Given		MLE(Std.)
Heritability of the simulated QTL is *H* ^*2*^ *=* 0.05																				
*r*	0.12		0.1329(0.0429)		0.22	0.2292(0.0148)		0.24		0.2611(0.0137)	0.2		0.2257(0.0084)		0.27		0.3077(0.0134)	0.3		0.3081(0.0108)
*k*_*1*_	0.003		0.0035(0.0035)		0.027	0.0272(0.0010)		0.09		0.083(0.0245)	0.015		0.0097(0.0028)		0.0018		0.0019(0.0002)	0.011		0.0109(0.0015)
*k*_*2*_	0.0006		0.0003(0.0009)		0.0011	0.0003(0.0006)		0.0007		0.0015(0.0017)	0.0009		0.0030(0.0008)		0.001		0.0018(0.0017)	0.0009		0.0007(0.0008)
*k*_*3*_	0.66		0.6547(0.0096)		0.69	0.6921(0.0104)		0.6		0.5970(0.0093)	0.55		0.5354(0.0105)		0.5		0.5054(0.0108)	0.65		0.6472(0.0105)
*k*_*4*_	0.55		0.5489(0.0079)		0.5	0.4938(0.0083)		0.6		0.6009(0.0081)	0.65		0.6679(0.0111)		0.6		0.5989(0.0087)	0.5		0.4973(0.0084)
Heritability of the simulated QTL is *H* ^*2*^ *=* 0.1																				
*r*	0.12		0.1378(0.0258)		0.22	0.2271(0.0118)		0.24		0.2517(0.0095)	0.2		0.2057(0.0179)		0.27		0.2969(0.0116)	0.3		0.3044(0.0093)
*k*_*1*_	0.003		0.0035(0.0020)		0.027	0.0267(0.0021)		0.09		0.0931(0.0122)	0.015		0.0106(0.0019)		0.0018		0.0020(0.0002)	0.011		0.0109(0.0012)
*k*_*2*_	0.0006		0.0004(0.0008)		0.0011	0.0005(0.0006)		0.0007		0.0013(0.0022)	0.0009		0.0025(0.0008)		0.001		0.0019(0.0016)	0.0009		0.0009(0.0010)
*k*_*3*_	0.66		0.6628(0.0074)		0.69	0.6884(0.0200)		0.6		0.5992(0.0062)	0.55		0.5387(0.0090)		0.5		0.5063(0.0143)	0.65		0.6502(0.0078)
*k*_*4*_	0.55		0.5361(0.0157)		0.5	0.4982(0.0077)		0.6		0.5994(0.0118)	0.65		0.6619(0.0070)		0.6		0.5969(0.0138)	0.5		0.4965(0.0183)
Heritability of the simulated QTL is *H* ^*2*^ *=* 0.2																				
*r*	0.12		0.1055(0.0092)		0.22	0.2253(0.0039)		0.24		0.2629(0.0216)	0.2		0.2059(0.0179)		0.27		0.2894(0.0069)	0.3		0.2939(0.0073)
*k*_*1*_	0.003		0.0035(0.0004)		0.027	0.0271(.0012)		0.09		0.0901(0.0153)	0.015		0.0108(0.0032)		0.0018		0.0019(0.0001)	0.011		0.0105(0.0008)
*k*_*2*_	0.0006		0.0005(0.0007)		0.0011	0.0009(0.0005)		0.0007		0.0010(0.0009)	0.0009		0.0024(0.0004)		0.001		0.0023(0.0012)	0.0009		0.0009(0.0007)
*k*_*3*_	0.66		0.6591(0.0052)		0.69	0.6899(0.0059)		0.6		0.6056(0.0086)	0.55		0.5521(0.0083)		0.5		0.5081(0.0128)	0.65		0.6477(0.0056)
*k*_*4*_	0.55		0.5464(0.0054)		0.5	0.4993(0.0057)		0.6		0.6017(0.0091)	0.65		0.6632(0.0052)		0.6		0.6020(0.0083)	0.5		0.5003(0.0062)
**Genetic Parameters**																				
		*H* ^*2*^ *=* 0.05					*H* ^*2*^ *=* 0.1					*H* ^*2*^ *=* 0.2								
		Given		MLE(Std.)			Given		MLE(Std.)			Given		MLE(Std.)						
*P*		0.6		0.6062(0.0216)			0.6		0.5962(0.0295)			0.6		0.5976(0.0243)						
*Q*_*1*_		0.4		0.3972(0.0211)			0.4		0.4026(0.0218)			0.4		0.4008(0.0251)						
*Q*_*2*_		0.5		0.5036(0.0223)			0.5		0.4963(0.0231)			0.5		0.4973(0.0272)						
*D*_*1*_		0.01		0.0152(0.0191)			0.01		0.0104(0.0177)			0.01		0.0114(0.0182)						
*D*_*2*_		0.02		0.0146(0.0213)			0.02		0.0172(0.0175)			0.02		0.0213(0.0203)						
**Matrix Structuring Parameters**																				
		*H* ^*2*^ *=* 0.05					*H* ^*2*^ *=* 0.1					*H* ^*2*^ *=* 0.2								
		Given		MLE(Std.)			Given		MLE(Std.)			Given		MLE(Std.)						
*ϕ*_*1*_		0.76		0.7400(0.0119)			0.7		0.6927(0.0089)			0.6		0.5978(0.0191)						
*ϕ*_*2*_		0.62		0.6161(0.0089)			0.564		0.5581(0.0099)			0.47		0.4667(0.0082)						
*φ*_*1*_		1.06		1.0521(0.0089)			1.0824		1.0782(0.0121)			1.11		1.1059(0.0055)						
*φ*_*2*_		1.19		1.1829(0.0086)			1.1757		1.1735(0.0094)			1.16		1.1565(0.0066)						
*ν*_*1*_		0.01		0.0108(0.0041)			0.01		0.0108(0.0022)			0.01		0.0107(0.0018)						
*ν*_*2*_		0.01		0.0103(0.0027)			0.01		0.0102(0.0018)			0.01		0.0101(0.0012)						
*ρ*		0.6624		0.6721(0.0183)			0.6624		0.6619(0.0072)			0.6624		0.6631(0.0084)						

Std. represents the standard deviation of the estimates obtained from 100 simulation replicates.

**Table 5:** MLEs of nonlinear ODE parameters that define the dynamics of cell transition for six different QTL genotypes and the association between the marker and QTL in a natural population of 400 based on different assumptions of heritability of the simulated QTL.

Cell Transition Parameters																										
	QQ						Qq				Qq+				qq				qq+				q+q+			
	Given			MLE(Std.)			Given	MLE(Std.)			Given			MLE(Std.)	Given		MLE(Std.)		Given	MLE(Std.)			Given			MLE(Std.)
Heritability of the simulated QTL is *H* ^*2*^ *=* 0.05																										
*r*	0.12			0.1321(0.0315)			0.22	0.2277(0.0122)			0.24			0.2711(0.0120)	0.2		0.2184(0.0075)		0.27	0.3049(0.0126)			0.3			0.3052(0.0096)
*k*_*1*_	0.003			0.0039(0.0028)			0.027	0.0270(0.0019)			0.09			0.0852(0.0123)	0.015		0.0103(0.0028)		0.0018	0.0020(0.0003)			0.011			0.0109(0.0018)
*k*_*2*_	0.0006			0.0005(0.0007)			0.0011	0.0013(0.0006)			0.0007			0.0014(0.0037)	0.0009		0.0028(0.0008)		0.001	0.0023(0.0016)			0.009			0.0074(0.0096)
*k*_*3*_	0.66			0.6578(0.0088)			0.69	0.6901(0.0204)			0.6			0.5985(0.0086)	0.55		0.5554(0.0096)		0.5	0.5094(0.0128)			0.65			0.6578(0.0122)
*k*_*4*_	0.55			0.5389(0.0085)			0.5	0.4963(0.0085)			0.6			0.6008(0.0114)	0.65		0.6658(0.0112)		0.6	0.5962(0.0097)			0.5			0.4957±0.0165)
Heritability of the simulated QTL is *H* ^*2*^ *=* 0.1																										
*r*	0.12			0.1338(0.0210)			0.22	0.2275(0.0112)			0.24			0.2516(0.0083)	0.2		0.2064(0.0151)		0.27	0.2949(0.0077)			0.3			0.3044(0.0076)
*k*_*1*_	0.003			0.0038(0.0015)			0.027	0.0269(0.0017)			0.09			0.0931(0.0087)	0.015		0.0104(0.0017)		0.0018	0.0020(0.0002)			0.011			0.0110(0.0013)
*k*_*2*_	0.0006			0.0006(0.0007)			0.0011	0.0015(0.0005)			0.0007			0.0014(0.0024)	0.0009		0.0026(0.0005)		0.001	0.0026(0.0015)			0.009			0.0062(0.0082)
*k*_*3*_	0.66			0.6630(0.0078)			0.69	0.6888(0.0198)			0.6			0.6014(0.0074)	0.55		0.5594(0.0082)		0.5	0.5082(0.0149)			0.65			0.6497(0.0137)
*k*_*4*_	0.55			0.5346(0.0208)			0.5	0.4985(0.0061)			0.6			0.5983(0.0131)	0.65		0.6626(0.0067)		0.6	0.5977(0.0125)			0.5			0.4957(0.0159)
Heritability of the simulated QTL is *H* ^*2*^ *=* 0.2																										
*r*	0.12			0.1247(0.0075)			0.22	0.2241(0.0031)			0.24			0.2735(0.0230)	0.2		0.2084(0.0146)		0.27	0.2873(0.0047)			0.3			0.2952(0.0062)
*k*_*1*_	0.003			0.0038(0.0002)			0.027	0.0270(0.0010)			0.09			0.105(0.0163)	0.015		0.0107(0.0314)		0.0018	0.0019(0.00001)			0.011			0.0107(0.0008)
*k*_*2*_	0.0006			0.0006(0.0005)			0.0011	0.0011(0.0005)			0.0007			0.0008(0.0013)	0.0009		0.0022(0.0003)		0.001	0.0023(0.0008)			0.009			0.0110(0.0073)
*k*_*3*_	0.66			0.6583(0.0063)			0.69	0.6878(0.0063)			0.6			0.6089(0.0110)	0.55		0.5531(0.0068)		0.5	0.5065(0.0072)			0.65			0.6484(0.0055)
*k*_*4*_	0.55			0.5471(0.0047)			0.5	0.5003(0.0054)			0.6			0.6053(0.0075)	0.65		0.6631(0.0036)		0.6	0.6010(0.0087)			0.5			0.5009(0.0062)
**Genetic Parameters**																										
		*H* ^*2*^ *=* 0.05							*H* ^*2*^ *=* 0.1							*H* ^*2*^ *=* 0.2										
		Given			MLE(Std.)				Given			MLE(Std.)				Given		MLE(Std.)								
*P*		0.6			0.5991(0.0178)				0.6			0.6025(0.5991)				0.6		0.0178(0.6025)								
*Q*_*1*_		0.4			0.4005(0.0181)				0.4			0.3996(0.4005)				0.4		0.0181(0.3996)								
*Q*_*2*_		0.5			0.5006(0.0187)				0.5			0.5007(0.5006)				0.5		0.0187(0.5007)								
*D*_*1*_		0.01			0.09(0.0121)				0.01			0.0093(0.09)				0.01		0.0121(0.0093)								
*D*_*2*_		0.02			0.0212(0.0126)				0.02			0.0202(0.0212)				0.02		0.0126(0.0202)								
**Matrix Structuring Parameters**																										
		*H* ^*2*^ *=* 0.05							*H* ^*2*^ *=* 0.1							*H* ^*2*^ *=* 0.2										
		Given			MLE(Std.)				Given			MLE(Std.)				Given		MLE(Std.)								
*ϕ*_*1*_		0.76			0.7504(0.0132)				0.7			0.6906(0.0068)				0.6		0.5975(0.0157)								
*ϕ*_*2*_		0.62			0.6206(0.0072)				0.564			0.5589(0.0068)				0.47		0.4657(0.0064)								
*φ*_*1*_		1.06			1.0598(0.0069)				1.0824			1.0801(0.0069)				1.11		1.1061(0.0044)								
*φ*_*2*_		1.19			1.1879(0.0056)				1.1757			1.1721(0.0106)				1.16		1.1551(0.0058)								
*ν*_*1*_		0.01			0.0107(0.0033)				0.01			0.0106(0.0021)				0.01		0.0105(0.0013)								
*ν*_*2*_		0.01			0.0104(0.0024)				0.01			0.0104(0.0012)				0.01		0.0101(0.0009)								
*ρ*		0.6624			0.6668(0.0152)				0.6624			0.6598(0.0130)				0.6624		0.6616(0.0108)								

Std. represents the standard deviation of the estimates obtained from 100 simulation replicates.

The simulation results of cell transition parameters, genetic parameters, and covariance-structural parameters with the linear and nonlinear model are shown in Tables 2 and 3 and Tables 4 and 5, respectively. The cell transition parameters can be estimated with high precision in both linear and nonlinear models. The precision of estimation of marker allele frequency is not affected by differences in heritability, but estimates of transition parameters, QTL allele frequency, and marker-QTL linkage disequilibrium are more precise for a higher than a lower heritability and for a bigger than a smaller population. The covariance-structural parameters can also be estimated, partly because of their simple structure for covariance modeling.

**Fig 3:**
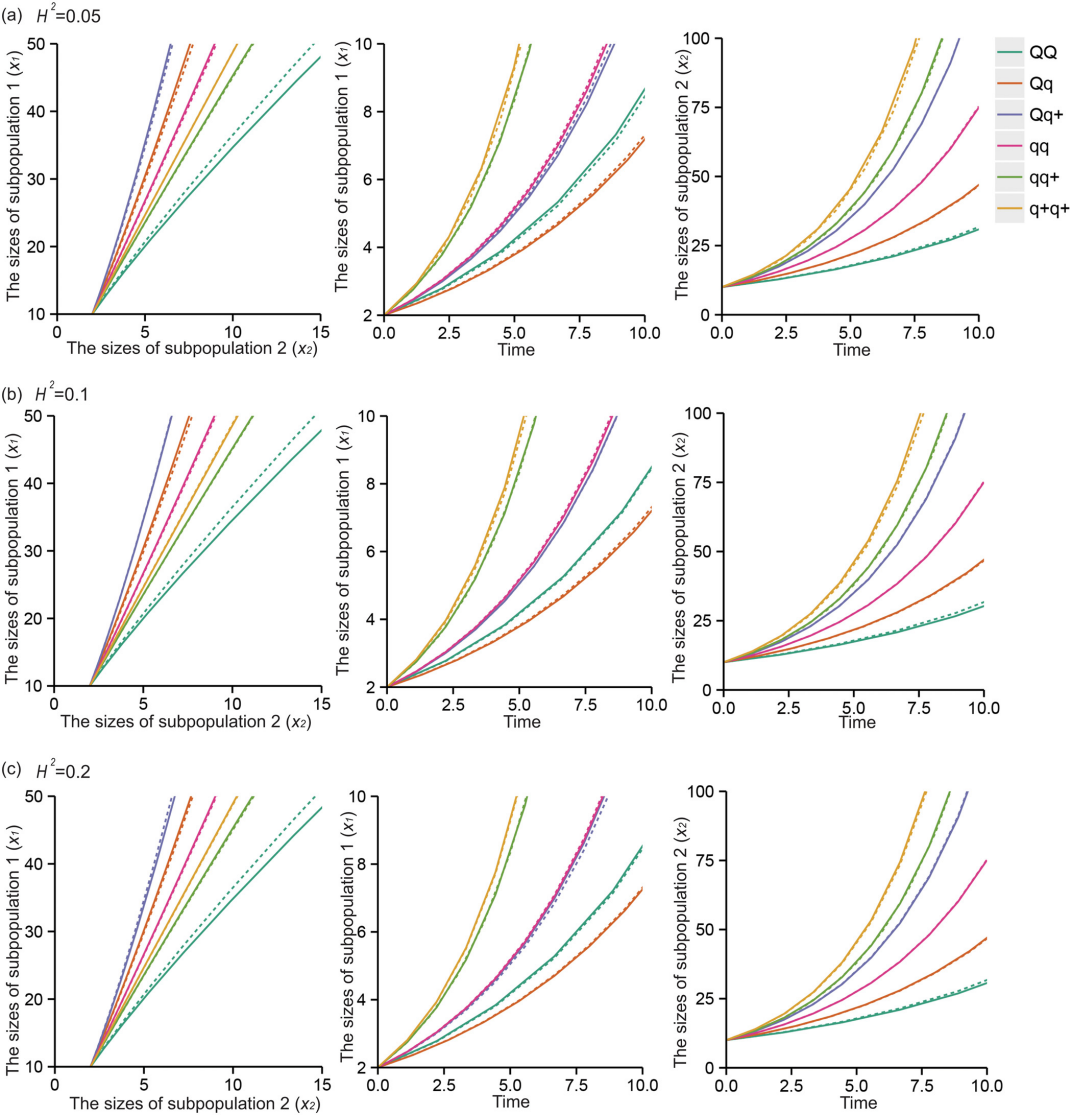
Estimated and true curves of systems mapping for the linear progenitor cell transition dynamics model. The curves show a putative QTL having six genotypes, *QQ*, *Qq*, *Qq* ^*+*^, *qq*, *qq* ^*+*^, and *q* ^*+*^ *q* ^*+*^, as indicated by colors in a natural population of 400 assuming heritability *H* ^*2*^ = 0.05 (a), *H* ^*2*^ = 0.1 (b), and *H* ^*2*^ = 0.2 (c), respectively. The broad consistency between the estimated (solid) and true curves (broken) suggests that the model provides a good estimate of the dynamic system.

**Fig 4:**
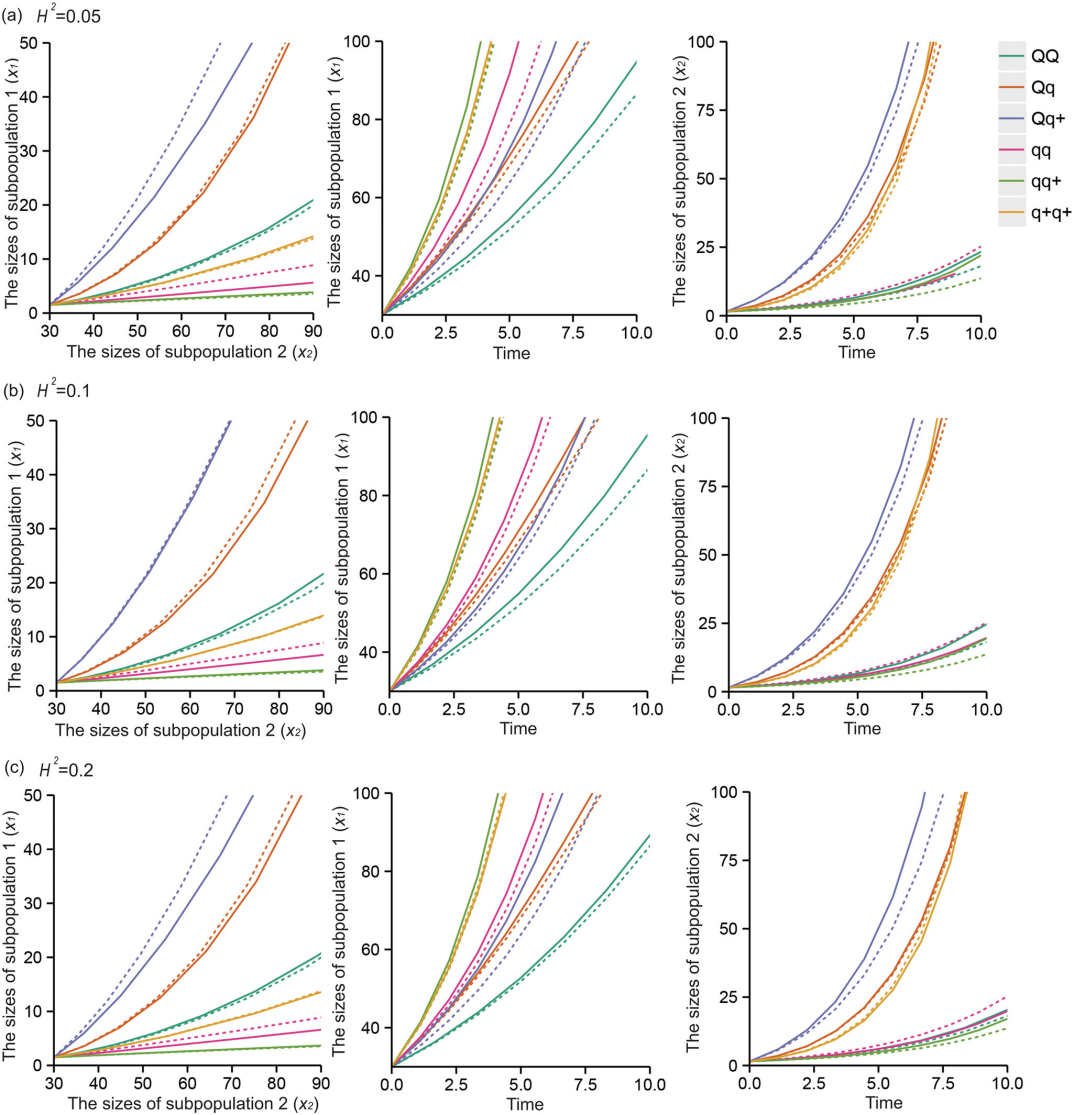
Estimated and true curves of systems mapping for the nonlinear progenitor cell transition dynamics model. The curves show a putative QTL having six genotypes, *QQ*, *Qq*, *Qq* ^*+*^, *qq*, *qq* ^*+*^, and *q* ^*+*^ *q* ^*+*^, as indicated by colors in a natural population of 400 assuming heritability *H* ^*2*^ = 0.05 (a), *H* ^*2*^ = 0.1 (b), and *H* ^*2*^ = 0.2 (c), respectively. The broad consistency between the estimated (solid) and true curves (broken) suggests that the model provides a good estimate of the dynamic system.

**Fig 5:**
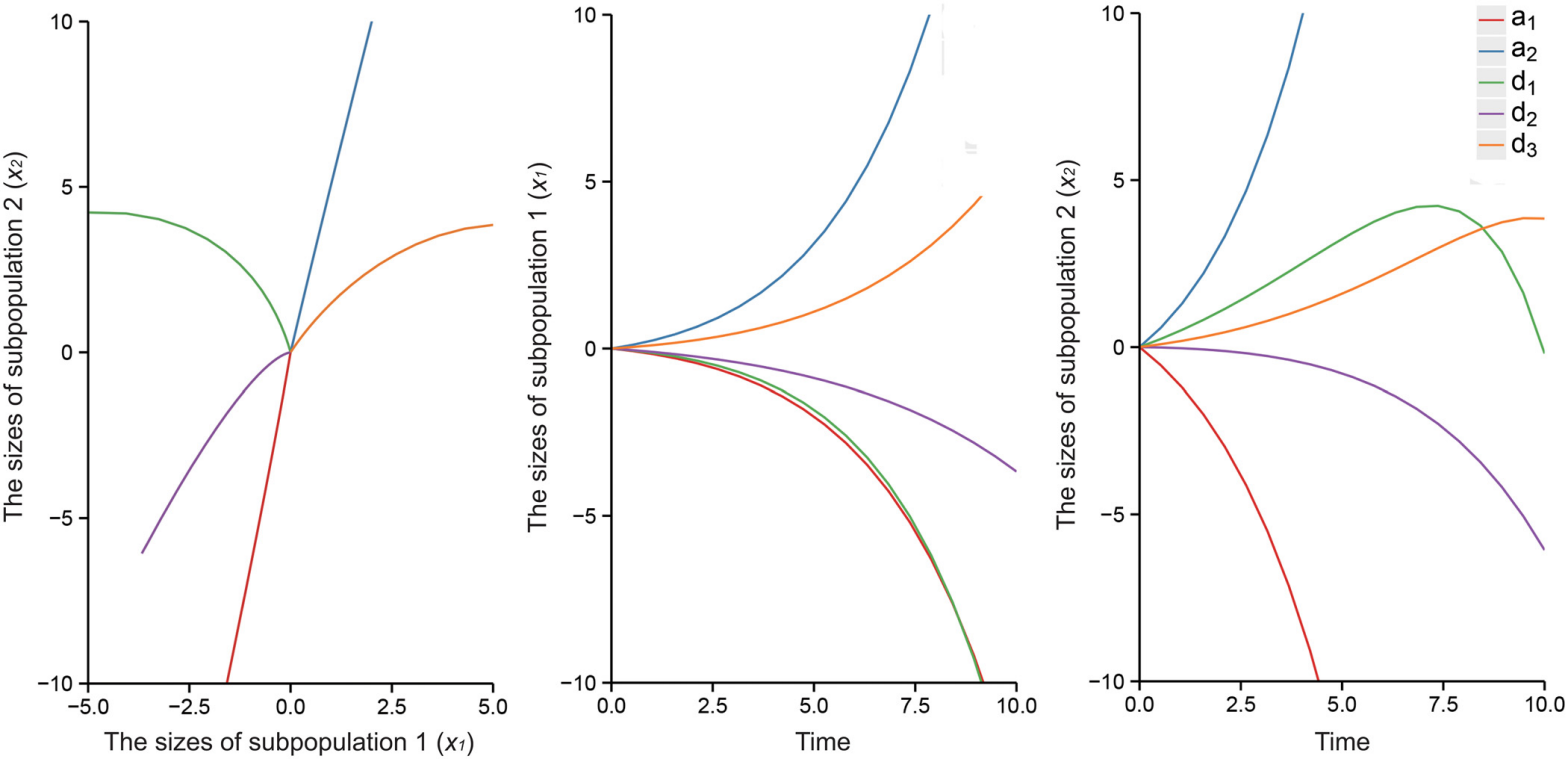
Trajectories of genetic effects on the linear progenitor cell transition dynamics model, including additive and dominant effects from *x*_*1*_ and *x*_*2*_.

**Fig 6:**
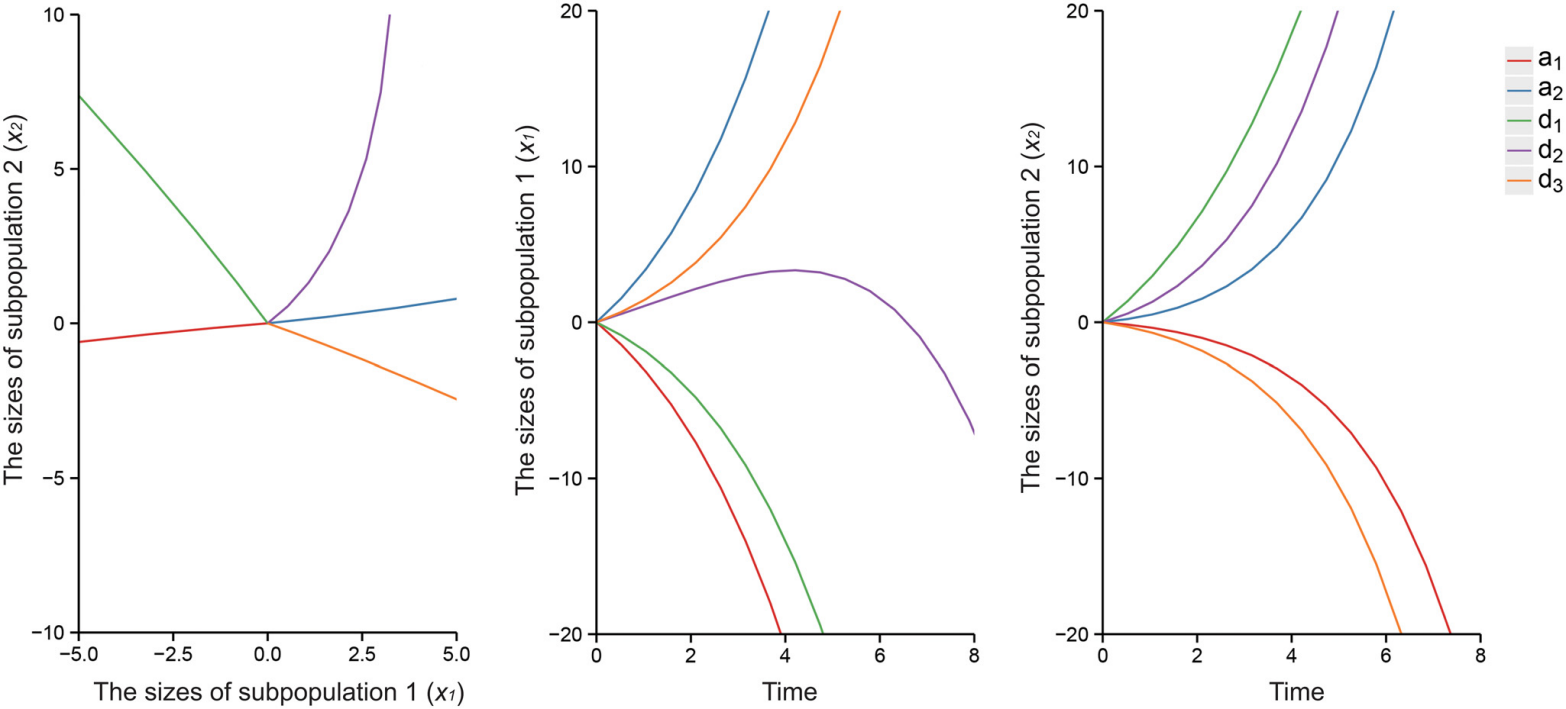
Trajectories of genetic effects on nonlinear progenitor cell transition dynamics model, including additive and dominant effects from *x*_*1*_ and *x*_*2*_.

The six QTL genotypes *QQ*, *Qq*, *Qq* ^*+*^, *qq*, *qq* ^*+*^, and *q* ^*+*^ *q* ^*+*^ are each hypothesized to have different response curves for different human populations. Figs 3 and 4 illustrate different forms of the cell population transition for six QTL genotypes, *QQ*, *Qq*, *Qq* ^*+*^, *qq*, *qq* ^*+*^, and *q* ^*+*^ *q* ^*+*^, under different heritabilities (0.05, 0.1 and 0.2) with size 400 populations, with the transitional values given in Table 3 for the linear model and Table 5 for the nonlinear model. Pronounced differences among the genotypes suggest that the QTL may affect cell transitions, resulting in different cellular phenotypes. Meanwhile, we considered the methylated QTL status (*Qq* ^*+*^, *qq* ^*+*^, and *q* ^*+*^ *q* ^*+*^), which could illustrate phenotypic differences among cells with the same DNA sequences. The cell transition values can be estimated from the model. The model displays great power in detecting a QTL responsible for cell transitions using the associated marker. The trajectories of additive and dominant effects of subpopulations *x*_*1*_ and *x*_*2*_ are shown as Figs 5 and 6 for the linear model and nonlinear model, respectively. We could observe the genetic architecture of the transition dynamics of the two subpopulation cells.

## Discussion

As more investigators use stem/progenitor cells to study the mechanisms of pluripotency and differentiation, they pay greater attention to cell heterogeneity and variability, which affects the use of pluripotent cells in regenerative medicine, disease modeling, and studying development processes. New methodologies, including single-cell and single-molecule analysis, as well as mathematical and computational modeling have been developed to explore pluripotent cell population heterogeneity. Emerging evidence has found differences in gene expression profiles at the mRNA level and functional differences in the differentiation within a heterogeneous cell population. However, the genetic status such as genetic backgrounds and epigenetic profiles including methylation of CpG islands and histone modifications remain unclear. Previous reports by Chang et al. [6] showed that transcriptome noise leads to clonal heterogeneity and controls lineage choices, but the genetic mechanisms of the heterogeneity and differentiation potential were not described.

In this article, we developed a statistical model that combined the mathematical concepts regarding cellular mechanisms and a general framework for mapping dynamic traits [15]. Both linear and nonlinear ODEs use specific parameters to describe how cells transit from one state to another during cell culture and test the magnitude and patterns of genetic effects in the process. The estimates of response curves are more precise for the linear model (Fig 3). However, the nonlinear model includes more parameters and information regarding complex cell proliferation and transition processes, so it may be more close to the true conditions. This model first considers methylated DNA as an allele in this “heterogeneity” based question, which indicates that different methylation states affect the phenotypes of the cells with the same genotype. Therefore, the model provides a tool to generate biologically meaningful hypotheses for understanding the genetic control of population cell heterogeneity.

The model proposed in this article considered only six genotypes; namely, *QQ*, *Qq*, *Qq* ^*+*^, *qq*, *qq* ^*+*^, and *q* ^*+*^ *q* ^*+*^, but biologically there should be more methylated states of the genotypes such as *Q* ^*+*^ *Q* ^*+*^, *Q* ^*+*^ *q*, and *Q* ^*+*^ *q* ^*+*^. The allele frequencies of the marker, QTL and their LD, joint marker and phenotypic data, and the statistical model will be more complex in this situation. In addition, further cellular and molecular experiments are required to confirm the exact DNA modification states of these cells, and a model incorporating multiple QTL and their interactive networks should be derived. However, the model will be useful in elucidating the genetic architecture, especially epigenetic mechanisms of cell heterogeneity, and could increase our understanding of the driving forces behind cell heterogeneity and transitions.
